# TRPV1-mediated presynaptic transmission in basolateral amygdala contributes to visceral hypersensitivity in adult rats with neonatal maternal deprivation

**DOI:** 10.1038/srep29026

**Published:** 2016-07-01

**Authors:** Ying Xiao, Xiaoqi Chen, Ping-An Zhang, Qiya Xu, Hang Zheng, Guang-Yin Xu

**Affiliations:** 1 Jiangsu Key Laboratory of Translational Research and Therapy for Neuro-Psycho-Diseases, Laboratory of Translational Pain Medicine, Institute of Neuroscience, Soochow University, Suzhou 215123, P.R. China; 2 Department of Gastroenterology, the First Affiliated Hospital of Henan College of Traditional Chinese Medicine, Zhengzhou 45000, P.R. China

## Abstract

The central mechanisms of visceral hypersensitivity remain largely unknown. It’s reported that there are highest densities of TRPV1 labeled neurons within basolateral amygdala (BLA). The aim of this study was to explore the role and mechanisms of TRPV1 in BLA in development of visceral hypersensitivity. Visceral hypersensitivity was induced by neonatal maternal deprivation (NMD) and was quantified by abdominal withdrawal reflex. Expression of TRPV1 was determined by Western blot. The synaptic transmission of neurons in BLA was recorded by patch clamping. It was found that the expression of TRPV1 in BLA was significantly upregulated in NMD rats; glutamatergic synaptic activities in BLA were increased in NMD rats; application of capsazepine (TRPV1 antagonist) decreased glutamatergic synaptic activities of BLA neurons in NMD slices through a presynaptic mechanism; application of capsaicin (TRPV1 agonist) increased glutamatergic synaptic activities of BLA neurons in control slices through presynaptic mechanism without affecting GABAergic synaptic activities; microinjecting capsazepine into BLA significantly increased colonic distension threshold both in control and NMD rats. Our data suggested that upregulation of TRPV1 in BLA contributes to visceral hypersensitivity of NMD rats through enhancing excitation of BLA, thus identifying a potential target for treatment of chronic visceral pain.

Irritable bowel syndrome is a functional gastrointestinal disorder characterized by chronic abdominal pain and altered bowel movements^1^. It is associated with psychosocial and genetic factors^2^,^3^. However, the exact causes of IBS are not clear, and there is a lack of effective therapeutics for the chronic visceral pain. Neonatal Maternal deprivation (NMD) is an animal model mimicking the effects of early life stress on the development of emotional and social behaviors. In addition, NMD induces visceral hypersensitivity in adult rats^4^,^5^,^6^. Therefore, the present study uses NMD as an animal model to mimic main pathophysiological features of IBS in human and to study the mechanisms of IBS in animals.

There has been a lot of evidence that IBS is related to the dysfunction of peripheral nervous system such as dosal root ganglion neurons. Studies in recent years show that the dysfunction of the brain is also underlying the mechanisms of IBS^7^. Up to date, knowledge of brain sites involved in IBS is limited. Since IBS is commonly comorbid with emotional responses such as depression and/or anxiety, many attentions are paid to amygdala which is a key forebrain structure underlying emotional responses^8^. Amygdala is constructed by several nuclei mainly including lateral nucleus (LA), basolateral (or basal) nucleus (BLA) and central nucleus (CeA)^9^. These nuclei are anatomically and functionally different from each other. CeA receives input from LA and BLA to form a circuitry. Somatic sensory information from the spinal dorsal horn is transmitted through the lateral thalamus to the cortical areas such as insular cortex and anterior cingulate cortex^10^, then to the amygdala, particularly the BLA^9^,^11^. In addition, CeA is also directly linked to nociceptive relay nuclei in the spinal cord and brain stem^11^. A growing body of evidence shows that the amygdala deals with pain-related information^1^,^9^,^11^,^12^,^13^,^14^.

Pain is known to be composed of sensory component and negative affective component. CeA is reported to play a role in negative affective component of visceral pain induced by intracolonic zymosan treatment^15^. CeA is also reported to be involved in enhanced sensory of visceral pain following repeated water avoidance stress^14^ or induced by corticotrophin-releasing factor 1 (CRF1) activation in CeA^16^ or by activation of glucocorticoid and mineralocorticoid receptors in CeA^1^,^17^. Since the laterocapsular part within CeA receives information of nociception coming from the LA and BLA which are important for attaching affective content to sensory information^9^,^18^,^19^,^20^, it is highly possible that BLA also contributes to sensory of visceral pain, which hasn’t been reported yet. However, the amygdala has differential stimulus-specific patterns of activation^11^. Tanimoto *et al*. reported that both BLA and CeA are involved in the negative affective component of chemical somatic pain induced by intraplantar injection of formalin, but only CeA is involved in the negative affective component of chemical visceral pain induced by intraperitoneal injection of acetic acid; neither CeA nor BLA mediates sensory components of those pains^11^. This suggests that the involvement of CeA in modulation of visceral sensory depends on the property of stimulus. NMD causes enhanced visceral pain induced by colorectal distention (CRD), and increases the activity of Na^+^/K^+^-ATPase in the amygdala^21^,^22^; the expression of BDNF and TrkB were increased in CeA after NMD^23^. It suggests that amygdala especially the CeA may take an important part in the visceral hypersensitivity after NMD. Although BLA is one of the upstream inputs of CeA, it still remains unclear whether BLA contributes to visceral hypersensitivity following NMD given the differences in activation between BLA and CeA.

Transient receptor potential vanilloid 1 (TRPV1) is a ligand-gated nonselective cation channel^24^,^25^. TRPV1 receptors are widely distributed in peripheral nervous systems such as dorsal root ganglion (DRG) neurons and colon, and are reported to be an important mediator in peripheral nervous systems in various types of pain, including neuropathic pain^26^,^27^, bone cancer pain^28^, inflammatory pain^29^,^30^ and visceral pain^31^,^32^,^33^,^34^,^35^,^36^. TRPV1 receptors are also widely expressed in the central nervous systems including periaqueductal gray (PAG)^37^, rostroventromedial medulla (RVM)^25^, cortex^38^,^39^, solitary tract nucleus^25^, insula^25^ and amygdala^15^,^39^,^40^. In the amygdala, there are highest densities of TRPV1 labeled neurons within BLA^40^. The endogenous agonist of the TRPV1 receptor, anandamide, can be synthesized within the BLA^40^,^41^. It is reported that TRPV1 in the brain interfers with the affective component of chemical and inflammatory spontaneous abdominal nocifensive responses and contributes to neuropathic pain in the limbic cortex^27^,^36^. However, the functional role of TRPV1 receptors in the BLA in visceral hypersensitivity following NMD is unclear.

In this study, we hypothesized that TRPV1 receptors in the BLA contribute to the visceral hypersensitivity in NMD rats. The expression of TRPV1 receptors in BLA was examined by Western blotting and qPCR technique from control and NMD rats. Changes in excitatory and inhibitory synaptic transmission were determined using patch-clamp recordings in coronal BLA slices from control and NMD rats. The expressing site of TRPV1 in BLA was proved by immunofluorescence study. The role of TRPV1 receptors in synaptic transmission and visceral hypersensitivity were explored pharmacologically.

## Results

### Enhanced expression of TRPV1 in BLA of NMD rats

**Figure 1:**
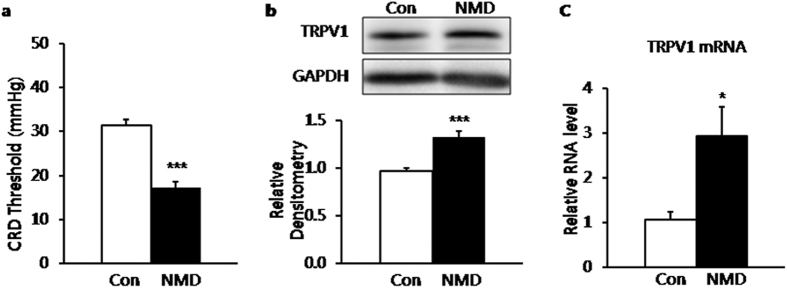
Visceral hypersensitivity and upregulation of TRPV1 in BLA of NMD rats. (**a**) A significant decrease in CRD threshold indicated visceral hypersensitivity of adult rats with NMD. (**b**) Expression of TRPV1 was significantly increased in BLA of NMD group. ***P < 0.001 vs. control. (**c**) mRNA level of TRPV1 was significantly increased in left BLA of NMD group. *P < 0.05 vs. control.

Chronic visceral hyperalgesia (CVH) was induced by NMD and determined by measuring the colorectal distention (CRD) threshold at the age 6–7 weeks. The CRD threshold was 31.35 ± 1.22 mmHg for control rats (Con, n = 14) and 17.26 ± 1.26 mmHg for NMD rats (n = 12). The CRD was significantly lower in NMD rats than in age-matched control rats (Fig. 1a, ***P = 3.31E-8, two-tailed two sample t-test). This result was consistent with the previous reports from our group^5^. Since it is reported that there are highest densities of TRPV1 labeled neurons within BLA^40^ and TRPV1 receptors are an important mediator in peripheral nervous systems in various types of pain^25^,^26^,^27^,^28^,^30^,^31^,^32^,^33^,^34^,^35^,^36^, western blotting assays were performed to determine whether expression of TRPV1 receptors was changed in BLA after NMD. Proteins isolated from BLA of control and NMD rats were probed with anti-TRPV1 antibody. Anti-TRPV1 antibody labeled a ~100 kDa molecular mass protein (Fig. 1b). Four weeks after NMD, the relative densitometry of TRPV1 was 1.27 ± 0.02 (n = 4). The relative densitometry of TRPV1 was 0.96 ± 0.03 (n = 3) for control group, indicating the upregulation of TRPV1 receptor in BLA after NMD (Fig. 1b, ***P = 0.0016, two-tailed two sample t-test). Meanwhile, the mRNA level of TRPV1 of left hemisphere was significantly increased in NMD group as well (Fig. 1c*P = 0.024, two-tailed two sample t-test). The relative mRNA level for control group is 1.06 ± 0.17(n = 4), while it is 2.92 ± 0.65 for NMD group(n = 3).

### Increase in glutamatergic synaptic activity of BLA neurons in NMD rats

**Figure 2:**
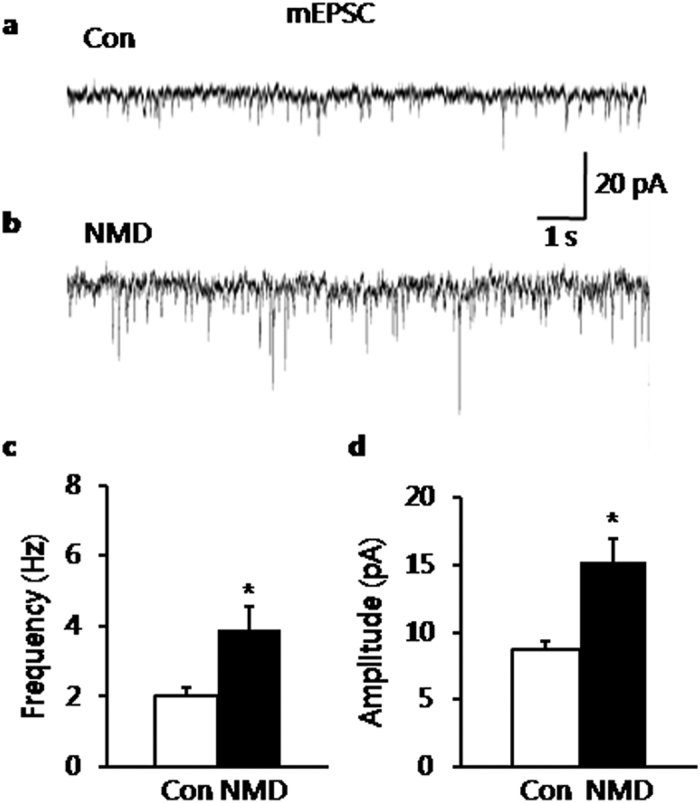
Increase in mEPSCs of neurons from NMD rats. (**a**) Recording illustrating mEPSCs of typical neurons in BLA slices of control rats. (**b**) Recording illustrating mEPSCs of typical neurons in BLA slices of NMD rats. (**c**) Increase of mEPSC frequency in NMD group. (**d**) Increase of mEPSC amplitude in NMD group. *P < 0.05 vs. Con.

Whether synaptic activity of BLA was altered by NMD was next determined by comparing miniature excitatory post-synaptic currents (mEPSCs) of BLA neurons between control and NMD group. The representative traces from two typical neurons of control and NMD slices illustrated an increase in frequency and amplitude of mEPSCs of BLA neuron in NMD group (Fig. 2a,b). The average results were also shown in Fig. 2c (n = 6 for each group, *P = 0.029, two-tailed two sample t-test) and Fig. 2d (n = 6 for each group, *P = 0.014, two-tailed two sample t-test). Mean frequency of mEPSCs averaged to 2.16 ± 0.20 Hz in control group and 3.92 ± 0.63 Hz in NMD group, respectively. In parallel, the mean peak amplitude of mEPSCs averaged to 9.11 ± 0.63 pA in control group and 14.47 ± 1.61 pA in NMD group, respectively. It suggested that the glutamatergic synaptic activity was enhanced in BLA of NMD rats.

### Increase in excitability of BLA neurons in NMD rats

**Figure 3:**
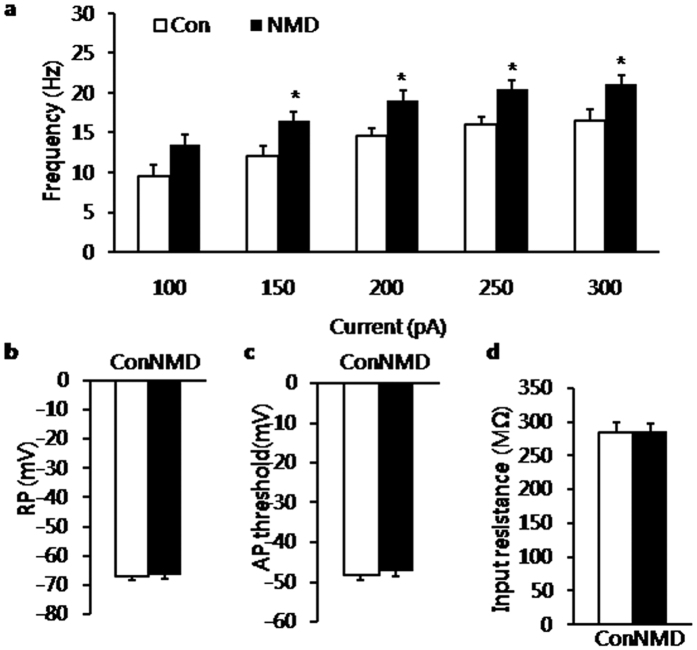
Membrane properties of pyramidal neurons in NMD rats. (**a**) Bar plot illustrating higher firing frequency of neurons in NMD rats than in control rats in response to different intensity of injected-currents. (**b**) Resting membrane potential was not altered after NMD. (**c**) Action potential (AP) threshold was not altered after NMD. (**d**) membrane input resistance was not altered after NMD. *P < 0.05 vs. control (Con).

The firing frequency of excitatory neurons of BLA was checked for control and NMD slices. Action potentials were evoked under current clamp mode by injecting intracellular depolarizing current with increasing intensity into BLA neurons. The neurons (n = 16) from NMD rats had a higher action potential firing rate than the neurons (n = 12) from control rats at 6–7 wk of age (Fig. 3a; *P* = 0.013, 0.011, 0.011, 0.03 for 150 pA, 200 pA, 250 pA, 300 pA, respectively; *P < 0.05 vs. Control, Mann-Whitney test for each intensity of current), which suggested the increased excitability of BLA excitatory neurons in NMD slices. However, there was no significant difference of resting membrane potential (−66.96 ± 1.05 mV for control; −66.47 ± 1.12 mV for NMD), action potential threshold (−48.24 ± 1.29 mV for control; −47.31 ± 1.31 mV for NMD) and membrane input resistance (284.33 ± 15.81 M Ω for control; 286.20 ± 11.75 M Ω for NMD) between two groups (Fig. 3b–d).

### Capsazepine decreased glutamatergic synaptic activity of BLA neurons in NMD group

**Figure 4:**
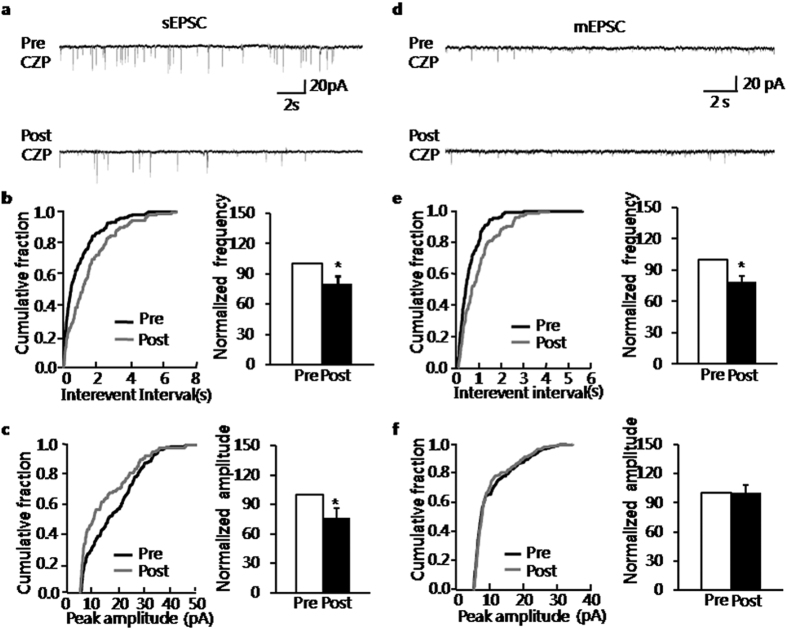
Capsazepine decreased glutamatergic synaptic activity of BLA in NMD slices through a presynaptic mechanism. (**a**) Representative traces illustrating sEPSCs before and after the addition of capsazepine (CZP, 5 μM). (**b**) Cumulative fraction of interevent intervals of sEPSCs in one BLA pyramidal neuron under the pre-drug and post-drug conditions (left); bar plot showing the significant decrease of sEPSC frequency by CZP (right); frequency of CZP-treated neuron was normalized by that from the same neuron before CZP-treatment. (**c**) Cumulative fraction of peak amplitude of sEPSCs under the pre-drug and post-drug conditions (left); bar plot showing the significant decrease of sEPSC amplitude by CZP (right); amplitude of CZP-treated neurons was normalized by that from the same neuron before CZP-treatment. (**d**) Representative traces illustrating mEPSCs before and after the addition of CZP at 5 μM. Na^+^ channel blocker TTX (1 μM) was used to block action potential firing. (**e**) Cumulative fraction of interevent intervals of mEPSCs in one BLA pyramidal neuron under the pre-drug and post-drug conditions (left); bar plot showing the significant decrease of mEPSC frequency by CZP (left); frequency of CZP-treated neurons was normalized by frequency from the same neuron before CZP-treatment. (**f**) Cumulative fraction of peak amplitude of mEPSCs under the before-drug and after-drug conditions (left); bar plot showing that mEPSC amplitude was unaffected by CZP treatment (right); amplitude of CZP-treated neuron was normalized by amplitude from the same neuron before CZP-treatment. *P < 0.05 vs. Pre.

Since TRPV1 receptors were upregulated and the excitability of BLA was increased in NMD group, we hypothesized that the increase of excitability of BLA in NMD group might be associated with the upregulation of TRPV1 receptors. Therefore, blockade of TRPV1 receptors was hypothesized to decrease excitability of BLA in NMD group. Capsazepine (CZP), an antagonist of TRPV1 receptors, was used in the present study to determine the effect of blocking TRPV1 receptors on spontaneous excitatory post-synaptic currents (sEPSCs) of excitatory neurons of BLA slices in NMD rats. A typical current trace illustrated the decrease in frequency and amplitude of sEPSCs in a neuron of BLA slices in NMD rats (Fig. 4a). It was also illustrated in the figures of cumulative fraction of inter-event intervals and peak amplitude for a representative neuron (Fig. 4b,c, left). Normalized mean values for frequency and amplitude were shown in Fig. 4b,c (right). It showed a significant decrease in average frequency from 6.14 ± 0.58 Hz before to 4.90 ± 0.54 Hz after the addition of CZP (5 μM), yielding a decrease to 80.64 ± 7.34% (Fig. 4b, n = 9, *P = 0.039, paired sample wilcoxon signed rank test). In parallel, the average mean amplitude of sEPSCs changed from 19.20 ± 1.98 pA without to 16.47 ± 2.09 pA with the presence of CZP, reflecting an decrease to 80.13 ± 8.52% (Fig. 4c, n = 9, *P = 0.031, paired sample wilcoxon signed rank test). It suggested that blockade of TRPV1 receptors decreased the spontaneous glutamatergic synaptic activity of BLA neurons in NMD rats.

### Presynaptic versus postsynaptic site of the action of capsazepine

To assess whether CZP acts on presynaptic or postsynaptic sites in the BLA, we used a well-established method, quantal analysis of mEPSCs. The frequency and amplitude of mEPSCs of BLA slices from NMD rats were measured before and after application of CZP (5 μM). The typical trace of a representative neuron showed that blockade of TRPV1 receptors by CZP decreased the frequency of mEPSCs recorded in BLA neurons of NMD rats (Fig. 4d). Kolmogorov–Smirnov test proved that CZP shifted the cumulative fraction of inter-event intervals of mEPSCs toward larger value (Fig. 4e, left) and decreased the mean frequency of mEPSC significantly (Fig. 4e, right, n = 6, *P = 0.015, paired sample wilcoxon signed rank test). There was a significant decrease in average frequency from 3.92 ± 0.63 Hz before, to 3.11 ± 0.56 Hz after the addition of CZP. The average decrease achieved 79.01 ± 5.56%. CZP had no significant effect on the amplitude of mEPSCs (Fig. 4f, paired sample wilcoxon signed rank test, *P* > 0.05). Mean amplitudes averaged to 14.47 ± 1.61 pA before and 14.24 ± 1.90 pA after addition of CZP (99.97 ± 8.73%, n = 6, *P* > 0.05, paired sample wilcoxon signed rank test). It is well known that change in frequency of mEPSCs reflects the presynaptic changes at the transmitter release site, whereas change in amplitude of mEPSCs reflects the changes at the postsynaptic membrane^13^,^41^. The result of mEPSCs suggested that the blockade site of TRPV1 receptors in NMD rats by CZP is most likely presynaptic.

### Capsaicin enhanced glutamatergic synaptic activity in BLA in control group

**Figure 5:**
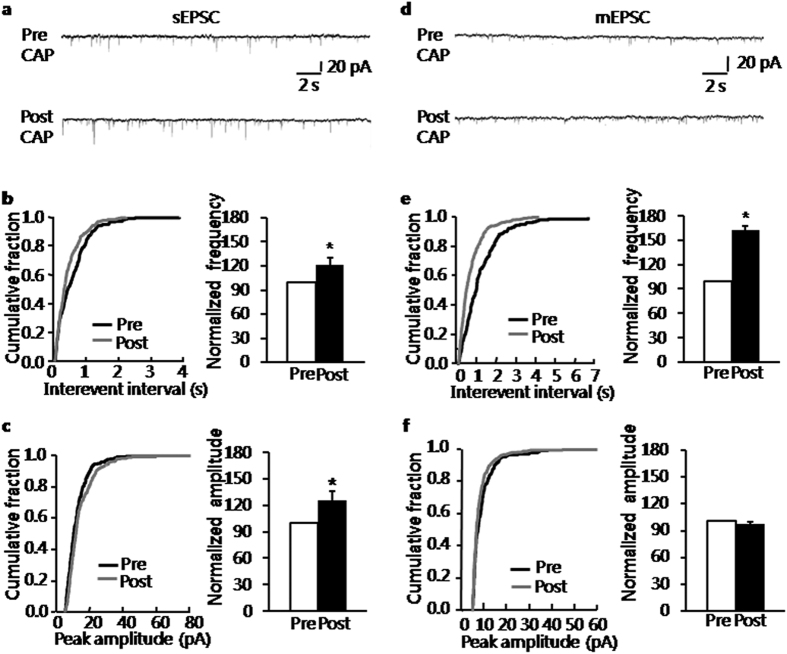
Enhancement of sEPSCs of neurons by capsaicin in control rats. (**a**) Representative traces illustrating sEPSCs before and after the addition of capsaicin (CAP, 5 μM). (**b**) Cumulative fraction of interevent intervals of sEPSCs in one BLA pyramidal neuron under the pre-drug and post-drug conditions (left); bar plot showing the significant increase in sEPSC frequency by CAP (right); frequency of CAP-treated neurons was normalized by that from the same neuron before CAP-treatment. (**c**) Cumulative fraction of peak amplitude of sEPSCs under the pre-drug and post-drug conditions (left); bar plot showing the significant increase of sEPSC amplitude by CAP (right); amplitude of CAP-treated neurons was normalized by that from the same neuron before CAP-treatment. (**d**) Representative traces illustrating mEPSCs before and after the addition of CAP at 5 μM; Na^+^ channel blocker TTX (1 μM) was used to block action potentials. (**e**) Cumulative fraction of interevent intervals of mEPSCs in one BLA pyramidal neuron under the pre-drug and post-drug conditions (left); bar plot showing the significant increase of mEPSC frequency by CAP (right); frequency of CAP-treated neurons was normalized by that from the same neuron before CAP-treatment. (**f**) Cumulative fraction of peak amplitude of mEPSCs under the pre-drug and post-drug conditions (left); bar plot showing that mEPSC amplitude was unaffected by CAP-treatment (right); amplitude of CAP-treated neuron was normalized by that from the same neuron before CAP-treatment. *P < 0.05 vs. Pre.

Since blockade of TRPV1 receptors decreased glutamatergic synaptic activity of BLA in NMD slices, activation of TRPV1 receptors by capsaicin (CAP) was hypothesized to increase the glutamatergic synaptic activity of BLA slices in control rats. The hypothesis was tested through recording sEPSCs before and after the addition of CAP in control slices. An increase of sEPSCs was observed after addition of CAP (5 μM). It showed that capsaicin was effective in increasing sEPSCs in 9 out of 11 pyramidal neurons tested. The representative current trace (Fig. 5a) demonstrated the increase in amplitude as well as the shortening of inter-event intervals with the presence of capsaicin in a typical neuron. It was also illustrated in the figures of cumulative fraction of inter-event intervals and peak amplitude (Fig. 5b,c, left). Normalized mean values for frequency and amplitude were shown in Fig. 5b,c (right). The average mean frequency of sEPSCs changed significantly from 4.86 ± 0.31 Hz before to 5.79 ± 0.70 Hz after the presence of CAP. The increase in frequency achieved 121.85 ± 8.91% (Fig. 5b, right, n = 11, *P = 0.042, paired sample wilcoxon signed rank test). In parallel, there was a significant increase in average amplitude of sEPSCs from 16.52 ± 1.66 pA, to 19.89 ± 1.37 pA. The increase in amplitude achieved 126.5 ± 9.84% (Fig. 5c, right, n = 11, *P = 0.032, paired sample wilcoxon signed rank test). In all cells, the sEPSCs were completely blocked with CNQX (10 μM), suggesting that sEPSCs were mediated by non-NMDA receptors. The effect of CAP was also tested on NMD group. The average mean frequency of sEPSCs of NMD group changed significantly from 5.89 ± 0.90 Hz before to 8.91 ± 1.93 Hz after the presence of CAP. The increase in frequency achieved 145.45 ± 12.98% (n = 6, *P* = 0.031, paired sample wilcoxon signed rank test). In parallel, there was a significant increase in average amplitude of sEPSCs of NMD group from 10.08 ± 0.85 pA, to 12.21 ± 0.94 pA. The increase in amplitude achieved 123.44 ± 9.42% (n = 6, *P* = 0.046, paired sample wilcoxon signed rank test). It is proved that CAP significantly increased both the frequency and amplitude of sEPSC of BLA neurons in NMD group, which was similar to the effect on control group. However, the CAP produced bigger effect on frequency of sEPSC comparing with the control group.

### Presynaptic versus postsynaptic site of the action of capsaicin

To assess whether CAP acts on presynaptic or postsynaptic sites in the BLA, the frequency and amplitude of mEPSCs were measured without and with the presence of CAP in brain slices of control rats. The representative current trace of a single neuron demonstrated that activation of TRPV1 receptors by CAP (5 μM) increased the frequency, but not amplitude, of mEPSCs recorded in BLA excitatory neurons (Fig. 5d). Kolmogorov–Smirnov test proved that capsaicin shifted the cumulative fraction of inter-event intervals of mEPSCs toward smaller values (Fig. 5e, left) and increased the mean mEPSC frequency significantly (Fig. 5e, right, n = 6, *P = 0.031, paired sample wilcoxon signed rank test). The average frequency increased from 2.16 ± 0.2 Hz without to 3.48 ± 0.27 Hz with the addition of CAP, yielding a rise to 162.74 ± 5.59%. CAP had no effect on the amplitude of mEPSCs (cumulative fraction of peak amplitude, *P* > 0.05, Kolmogorov–Smirnov test, Fig. 5f). The present result of mEPSCs suggested that the site of activation of TRPV1 receptors by CAP is presynaptic in control slices, which is consistent with the site of blockade of TRPV1 receptors in BLA slices of NMD rats.

### Capsaicin did not affect GABAergic synaptic activity in BLA neurons of control group

**Figure 6:**
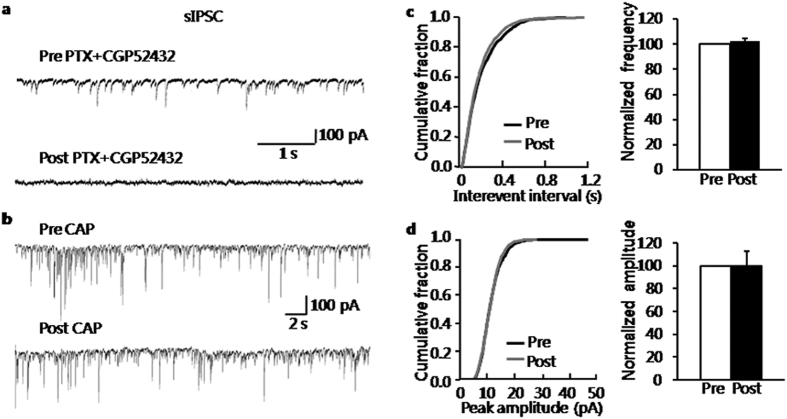
Capsaicin did not affect sIPSCs in BLA neurons of control rats. (**a**) Representative traces illustrating sIPSCs were eliminated after the addition of picrotoxin (PTX, 100 μM) and CGP52432 (2 μM). Applications of glutamatergic receptor antagonists CNQX (10 μM) and D-AP5 (30 μM) were used in the external solution to block excitatory synaptic transmission. (**b**) Recordings illustrating sIPSCs before and after the addition of CAP (5 μM). (**c**) Cumulative fraction of interevent intervals of sIPSCs in one BLA neuron under the pre-drug and post-drug conditions (left); bar plot showing that frequency of sIPSCs was not altered by CAP (right); frequency of CAP-treated neurons was normalized by that from the same neuron before CAP-treatment. (**d**) Cumulative fraction of peak amplitude of sIPSCs under the pre-drug and post-drug conditions (left); bar plot showing that amplitude of sIPSCs was not changed by CAP (right); amplitude of CAP-treated neuron was normalized by that from the same neuron before CAP-treatment.

Since there are inhibitory interneurons in the BLA, the inhibitory synaptic transmission in BLA was next characterized before and after the addition of CAP (5 μM). The typical trace of spontaneous inhibitory post-synaptic currents (sIPSCs) of a representative neuron was shown in Fig. 6a (upper trace). The sIPSCs were blocked by the mixture of picrotoxin (PTX, 100 μM) and CGP52432 (2 μM, Fig. 6a, lower trace), suggesting that sIPSCs were mediated by GABAergic receptors. The traces of sIPSCs of a typical neuron before and after the addition of CAP were shown in Fig. 6b. Kolmogorov–Smirnov test proved that CAP did not affect the cumulative fraction of either inter-event interval or peak amplitude of sIPSCs of a representative neuron in control slices (Fig. 6c,d, left). The average results from 6 recorded neurons were also shown in Fig. 6c,d (right). The frequency averaged to 12.92 ± 1.6 Hz before and 13.21 ± 1.6 Hz after the addition of CAP, with an increase in frequency of control slices averaging to 102.35 ± 2.41% (n = 6, *P* > 0.05). Meanwhile, mean control amplitudes averaged to 67.02 ± 22.98 pA before and 61.11 ± 19.83 pA after addition of CAP, with an increase in peak amplitude of control slices averaged to 100.27 ± 12.75% (Fig. 6d, n = 6, *P* > 0.05).

### Expression of TRPV1 receptors in BLA

**Figure 7:**
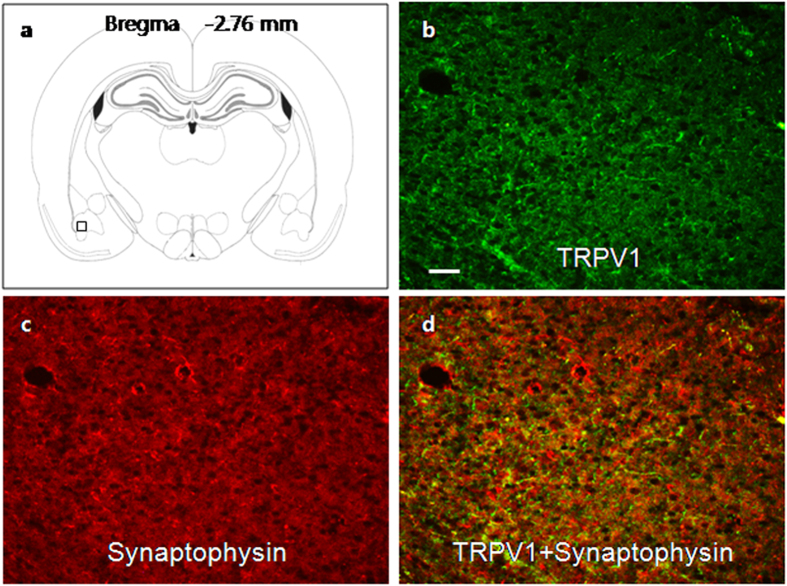
Colocalization of TRPV1 receptors and synaptophsin. (**a**) Illustration of BLA area (a small black square in the bottom left) was examined for immunostain. (**b**) TRPV1 receptors are marked by green dots. (**c**) Synaptophsin proteins are marked by red dots. (**d**) Merge of TRPV1 and synaptophsin. Scale bar in figure b equals100 μm.

We next verified the location of TRPV1 receptors in BLA by immunofluorescence study. Since synaptophsin is a widely accepted presynaptic vesicle protein, its antibody was used in the study to mark the presynaptic sites^42^. As indicated in Fig. 7, many TRPV1 receptors (Fig. 7b) were colocalized with synaptophsin (Fig. 7c,d). The result is consistent with the presynaptic mechanism of TRPV1 receptors as suggested by our electrophysiological studies.

### Alleviation of visceral pain in NMD and control rats by capsazepine treatment

**Figure 8:**
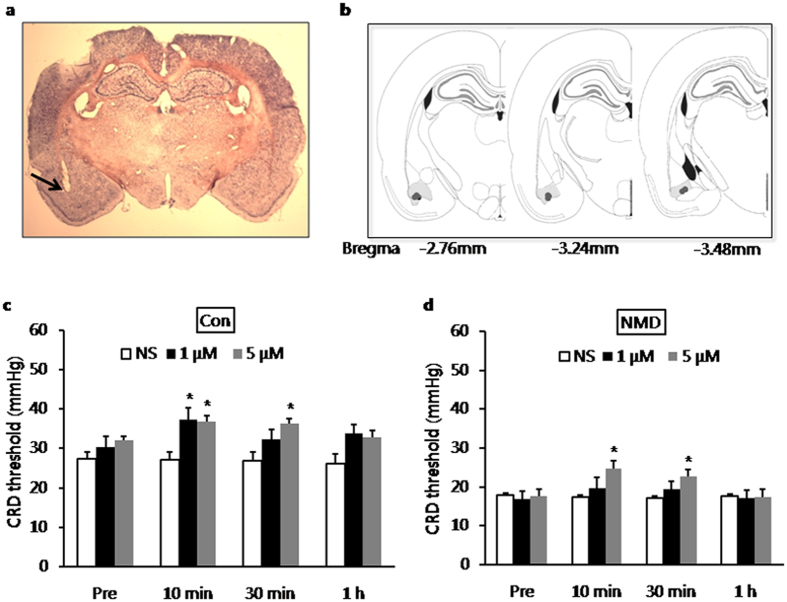
Increase in CRD threshold by microinjection of capsazepine into BLA. (**a**) A representative picture of actual track of microinjection in the BLA of left hemisphere as pointed out by the black arrow. (**b**) The schematic drawings of injection sites in the BLA as marked out by the black circles. (**c**) Bar plot illustrating an increase in CRD threshold in control rats by 1 μM CZP when tested 10 min after microinjection and also by 5 μM CZP when tested 10 min and 30 min after microinjection of the drug. (**d**) Bar plot illustrating an increase in CRD threshold in NMD rats by 5 μM CZP when tested 10 min and 30 min after microinjection of the drug. *P < 0.05 vs. normal saline (NS) for each time point.

We next examined the effect of microinjection of CZP into BLA on visceral sensitivity of NMD and age-matched control rats. The injection sites were verified histochemically as shown in Fig. 8a. Illustrations of injection sites in BLA were shown in Fig. 8b. The effect of CZP on CRD threshold was examined in NMD rats before and 10 min, 30 min and 1 hour after CZP injection. CZP was administrated stereotactically into BLA of left hemisphere and normal saline (NS, 0.9% NaCl) was injected in the same volume (1 μl) as control. Microinjection of CZP significantly increased CRD threshold in NMD rats at concentration of 5 μM (Fig. 8d, n = 6 for NS group; n = 5 for 1 μM group; n = 7 for 5 μM group; At 10 min post-injection: CRD threshold was 17.22 ± 0.60 mmHg, 19.53 ± 2.94 mmHg, 24.64 ± 1.96 mmHg for NS group, 1 μM group and 5 μM group, respectively; *P = 0.035 for 5 μM vs. NS, Tukey post-hoc test following One-way ANOVA, *P = 0.038 for One-way ANOVA; At 30 min post-injection: CRD threshold was 17.00 ± 0.63 mmHg, 19.23 ± 2.08 mmHg, 22.66 ± 1.61 mmHg for NS group, 1 μM group and 5 μM group, respectively; *P = 0.003 for 5 μM vs. NS, Mann-Whitney test as post hoc analysis following Kruskal-Wallis ANOVA, *P = 0.014 for Kruskal-Wallis ANOVA). However, injection of CZP didn’t have any effect on CRD threshold of NMD rats at concentration of 1 μM. The effect of 5 μM CZP disappeared when tested at 1 hour post-injection. To test whether the drug effect was confined only to NMD rats, the effect of CZP on CRD threshold was also examined in control rats at the same time points as did to the NMD rats. Both 1 μM and 5 μM of CZP (1 μl) significantly increased CRD threshold in control rats, with the longer duration of medication at the concentration of 5 μM. The effect of CZP also disappeared when tested at 1 hour after drug injection (Fig. 8c, n = 7 for NS group; n = 6 for 1 μM group; n = 8 for 5 μM group; At 10 min post-injection: CRD threshold was 27.07 ± 1.98 mmHg, 37.25 ± 3.09 mmHg, 36.66 ± 1.72 mmHg for NS group, 1 μM group and 5 μM group, respectively; *P = 0.018 for 1 μM vs. NS, *P = 0.006 for 5 μM vs. NS, Mann-Whitney test as post hoc analysis following Kruskal-Wallis ANOVA, *P = 0.009 for Kruskal-Wallis ANOVA; At 30 min post-injection: CRD threshold was 26.79 ± 2.33 mmHg, 32.40 ± 2.40 mmHg, 36.21 ± 1.44 mmHg for NS group, 1 μM group and 5 μM group, respectively; *P = 0.007 for 5 μM vs. NS, Tukey post-hoc test following One-way ANOVA, *P = 0.009 for One-way ANOVA). Injection itself didn’t have any effect on CRD threshold as demonstrated by the data of NS for both groups. These data proved that unilateral blockaded of TRPV1 receptors in BLA alleviated the visceral hypersensitivity to CRD under both normal and pathological conditions. However, the NMD rats needed more TRPV1 antagonist to take effect, which was consistent with the upregulation of TRPV1 receptors in NMD rats.

## Discussion

The present study demonstrated the role of TRPV1 in the BLA underlying chronic visceral hypersensitivity in adult rats with NMD. It was proved that NMD enhanced expression of TRPV1, which participated in the increased excitation of BLA neurons, eventually resulting in visceral hypersensitivity in adult rats.

A growing body of evidence showed that the generation and enhancement of pain responses can be modulated by amygdala; both CeA and BLA can modulate negative affective component of pain^11^,^15^; CeA can modulate sensory component of pain^1^,^14^,^16^,^17^. However, to the best our knowledge, there has no report about the modulatory role of BLA in sensory component of pain although BLA is one of the inputs to CeA. It was proved in the present study that microinjection of capsazepine into unilateral BLA increased the CRD threshold under both physiological and pathological conditions. Since blockade of TRPV1 receptors in BLA decreased the excitation of neurons in BLA, it suggested that decreasing the excitation of BLA could alleviate visceral pain in response to CRD. Therefore, the present study added new evidence that BLA can modulate sensory component of visceral pain in response to CRD. It is known that BLA does not take part in chemical visceral pain induced by intraperitoneal injection of acetic acid^11^. Combining our findings, it suggested that the involvement of BLA in modulation of visceral sensory depends on the property of stimulus, which is similar to the manner of CeA action.

In terms of the role of central TRPV1 in visceral pain, it is reported that TRPV1 receptors modulate the affective component of chemical and inflammatory spontaneous abdominal nociceptive responses and contribute to neuropathic pain in the limbic cortex^27^,^36^. The present study proved that TRPV1 receptors in BLA interfered with the visceral sensory in response to CRD and contributed to the visceral hypersensitivity of rat with NMD. The conclusion is based on the following observations: ①Unilateral microinjection of TRPV1 antagonist into BLA increased the CRD threshold that elicited an observable abdominal withdrawal reflex (Fig. 8); ② Both the expression of TRPV1 in BLA and mRNA level of TRPV1 in left BLA were increased in NMD rats (Fig. 1); ③ It needed higher effective dose of TRPV1 antagonist in NMD rats than in control rats to alleviate visceral pain (Fig. 8). In contrast, stimulation of TRPV1 in the PAG has been proved to inhibit pain by either acting on the downstream rostral ventromedial neurons that mediate analgesia or by desensitizing the activity of other neurons involved in inducing hyperalgesia^25^. This suggested that the modulatory effect on pain by activation of TRPV1 in the brain depends on which brain areas are activated by TRPV1 receptors. That microinjection of TRPV1 antagonist into left BLA alleviated visceral pain also suggested that left BLA might play a major role in the development of visceral hypersensitivity induced by NMD, which is in consistent with the more active role of left amygdala in emotional processing^43^,^44^.

In the present study, the increase in frequency of mEPSCs by capsaicin (Fig. 5), without significant change in amplitude of mEPSCs, strongly suggested that these effects were attributable to the action at presynaptic site of BLA, which was consistent with the result from immunofluorescence study (Fig. 7); meanwhile, GABAergic synaptic transmission was not affected by stimulation of TRPV1 receptors in the BLA (Fig. 6). These are in agreement with that obtained from the spinal cord^45^,^46^, the locus ceruleus^47^ and the substantia nigra^10^. That the excitability of neurons of BLA was dramatically increased in NMD group without changing the resting membrane potential, action potential threshold and input resistance could also be attributed by the presynaptic action of capsaicin (Fig. 3). Moreover, the bigger effect of CAP on frequency of sEPSC of NMD group could also be attributed by upregulation of TRPV1 receptors at the presynaptic site. However, being different from those mentioned structures of central nervous system, capsaicin neither affected frequency nor amplitude of mEPSCs or mIPSCs in LA projection neurons^40^. Taken together, activation of TRPV1 by endogenous factors such as anandamide can control glutamate neurotransmission in the BLA without any effect on GABA release. Since the activation of TRPV1 receptors increased glutamate neurotransmission, upregulation of TRPV1 receptors contributed to the over excitation of BLA in NMD rats (Fig. 2). Although TRPV1 was proved to take effect presynapticly, the amplitude of mEPSC of BLA was significantly increased by NMD as well as the frequency of mEPSC when compared to those of control group. It suggested that the expression or insertion of AMPARs on the membrane of postsynaptic site might be enhanced. Since it is reported that enhancement of CaMKII signaling in BLA increases Glu/AMPA receptor subunit A1 expression, which facilitates anxiety-like behavior and hyperactivation of the amygdala^48^, the increase in the amplitude of mEPSC of NMD rats might be caused by the enhancement of CaMKII signaling in BLA.

Besides to upregulation of TRPV1, there might be several other modulators contributing to the activation of BLA in NMD rats. These include elevated corticosterone level and increment in corticotropin-releasing factor (CRF) immunoreactive neurons^8^,^47^. Since the upregulation of CRF in CeA following repeated exposure to water avoidance stress accompanied the increase in visceral sensitivity to CRD^49^, the role of CRF in BLA maybe similar to its role in CeA. NMD caused upregulation of brain-derived neurotrophic factor and its receptor TrkB in the CeA and p-Erk expression in the amygdala^23^. It is also possible that the BDNF and TrkB expression was upregulated in BLA, although these hypotheses still lack direct evidence. In addition to amygdala, there are several other parts of central nervous system taking part in modulation of visceral pain. Anterior cingulate cortex mediates visceral pain, and there are direct and indirect reciprocal connections between ACC and amygdala^10^,^49^,^50^. Furthermore, it is reported that the amygdala-mediated colorectal hypersensitivity involves sensitization of spinal neurons through amygdala-periaqueductal gray-rostroventromedial medulla-spinal cord pathway-dependent descending facilitation^51^,^52^.

In conclusion, the present study showed that the upregulation of TRPV1 in BLA participates in visceral hypersensitivity of NMD rats through a presynaptic mechanism by increasing glutamate neurotransmission and sensitizing the BLA neurons. Application of TRPV1 antagonists in BLA can alleviate the visceral pain caused by CRD in both physiological and pathological conditions. The present results provide new insights into the central mechanisms of chronic visceral pain in IBS patients.

## Materials and Methods

### Induction of chronic visceral hyperalgesia (CVH)

CVH was induced by NMD, as described previously^53^. In brief, from postnatal days (PND) 2 to 15 male pups of Sprague-Dawley rats for the NMD group were placed in isolated cages with an electric blanket underneath the cage for 3 hours daily. When the separation finished, pups were back to their dams. Pups for the control group were kept with the dam in cages without handling. On PND 21, pups were weaned and kept without their dam. Experiments began at 6–7 weeks of age. All rats were maintained on a 12 h light/dark cycle and with free access to food and water. Animal care and handling of the animals were approved by the Institutional Animal Care and Use Committee at Soochow University and were strictly in accordance with the guidelines of the International Association for the Study of Pain.

### Measurement of colorectal distention (CRD) threshold

Visceral hypersensitivity was assessed by CRD threshold as described previously^54^. Briefly, a flexible balloon (4 cm) was inserted 6 cm into the colon and rectum via the anus after the rats were lightly anesthetized with isofluorane. The balloon was made of a surgical glove finger attached to a tygon tubing. The tubing was fixed to the tail with a tape. Rats were allowed to recover for 30 minutes in small isolated cubicles of lucite before CRD was performed. The balloon was slowly inflated using a sphygmomanometer until an observable abdominal withdrawal reflex of tested rats was seen by an observer in a blinded manner. Meanwhile, the distention pressure was read from the sphygmomanometer at the stop time and was used as CRD threshold. The measurement was repeated three times with at least 2 minute interval for recovery.

### Drug administration

For behavioral experiments, capsazepine (CZP) or normal saline (NS) was stereotactically injected into the unilateral BLA of rats. Since NMD mimicking the effects of early life stress on the development of emotional and social behaviors and left amygdala is more often activated than the right amygdala in emotional processing^43^,^44^, drug was injected into left BLA. In order to minimize tissue damage, one stainless guide cannula with 24 gauge were fixed unilaterally on the left hemisphere of the skull aiming at BLA (coordinates with respect to Bregma: AP −2.92 mm, ML 5.0 mm, DV 8.4 mm, angle 0) using dental cement. The cannula (exposed on the skull surface for 4 mm) was closed with a curved 26.5 gauge stainless steel injector needle head to prevent blockade. The rats were allowed to recover for at least 3–4 days from the surgery before behavioral tests. Ten minutes before performing behavioral tests, the curved needle heads were removed from the guide cannula; CZP (diluted by isotonic saline (0.9% wt/vol NaCl)) or isotonic saline (1 μl) were injected into BLA of left hemisphere by a 26.5 gauge needle head through the guide cannula and reached a final depth of 8.8 mm below dura. The microinjection lasted over 2 minutes through the cannula and the needle head was left for an additional 2 minutes to minimize back flow. CRD was performed before drug microinjection and at 10, 30 and 60 minutes after microinjection. The drug concentrations used in the study were based on our preliminary study and previous work in our lab^33^.

### Western blotting

Acute dissecting tissue of BLA of both hemispheres was prepared in ice-cold, oxygenated fresh ACSF, containing (in mM): 130 NaCl, 5 KCl, 2 KH_2_PO_4_, 10 HEPES, 10 Glucose, 6 MgSO_4_ and 1.5 CaCl_2_, pH 7.2 (adjusted osmolarity with sucrose to 305 mosM). Since the external capsule can be used as a landmark to define the borders of the lateral and basolateral amygdala; meanwhile, hippocampus can be used as a landmark to define the position of the sections relative to Bregma (approximately −2.5 ~ −3.5 mm relative to Bregma according to the Rat Brain in Stereotaxic Coordinates (6^th^ edition, 2007)); basolateral amygdala can be distinguished with other areas around as a darker area and be accurately dissected out from 1 mm coronal sections. After fractionating of BLA protein extract on 4% and 10% polyacrylamide gels, proteins were transferred to polyvinylidene difluoride membranes. For TRPV1, membranes of 70–130KD and 35–55kD were then blocked in Tris-buffered saline (TBS) under room temperature containing 5% dilution of non-fat milk powder. Membrane of 70–130KD was incubated with anti-TRPV1 antibody (1:200, Alomone labs, ACC-030) and the membrane of 35–55kD was incubated with anti-GAPDH antibody (1:200, SANTA CRUZ, sc-25778) under 4 °C overnight in TBS containing 1% milk. After washed in TBS containing 0.5% Tween-20 (TBST), membranes were incubated with horseradish peroxidase-conjugated secondary antibodies in TBS containing 1% milk at room temperature. Bands of films were visualized using an enhanced chemiluminescene detection kit for HRP (EZ-ECL, Biological Industries, 20-500-120) and appropriate exposure to chemiluminescent imaging system (ChemiDoc XRS, Biorad). Band intensities were measured using Image J software. TRPV1 protein expression was normalized to GAPDH.

### Real-time quantitative polymerase chain reaction for TRPV1 mRNA

Since NMD mimicking the effects of early life stress on the development of emotional and social behaviors and left amygdala is more often activated than the right amygdala in emotional processing^43^,^44^, total RNA was extracted from BLA of left hemisphere from control and NMD rats with Trizol (ambion, 15596026). cDNA was synthesized from total RNA using an Reverse transcription kit (TRANSGEN BIOTECH, AE301-03) following the supplier’s instructions. The sequences of the primer pairs for trpv1 used in quantitative polymerase chain reaction are: 5′ TACCCGGCTTTTTGGGAAGG3′ (F) and 5′ CTCTGAGCCACAGCATCGAA3′(R); the sequence of the primer pairs for gapdh (as an internal control) used in quantitative polymerase chain reaction are: 5′ TGGAGTCTACTGGCGTCTT 3′ (F) and 5′ TGTCATATTTCTCGTGGTTCA 3′(R).

Control reactions were performed in the absence of cDNA templates. The relative expression level for trpv1 gene was normalized by the Ct value of gapdh using a 2^−ΔΔCt^ relative quantification method.

### Slice preparation

Rats of both control and NMD groups (100–130 g) were decapitated after deep anesthesia with 4% chloral hydrate. The brain was rapidly removed and embeded with 1.6% high strength agarose (type I-B, Sigma, USA). The agarose block with brain tissue was placed into 32 °C oxygenated (95% O_2_, 5% CO_2_) NMDG-based solution with the following composition (in mM): 93 NMDG, 2.5 KCl, 1.2 NaH_2_PO_4_, 30 NaHCO_3_, 20 HEPES, 5 sodium ascorbate, 2 Thiourea, 3 sodium pyruvate, 12 NAC, 10 MgSO_4_, 0.5 CaCl_2_ and 25 glucose, according to Ting *et al*.^55^. Coronal slices (400 μm) were cut with vibroslicer VF-300 (Precisionary Instruments Inc.) and put into 32 °C NMDG-based solution to recover for 10 minutes, then left to adapt to room temperature in oxygenated holding solution with the following composition (in mM): 94 NaCl, 2.5 KCl, 1.2 NaH_2_PO_4_, 30 NaHCO_3_, 20 HEPES, 5 sodium ascorbate, 2 Thiourea, 3 sodium pyruvate, 2 MgSO_4_, 2 CaCl_2_, 12 NAC and 25 glucose. The slices were then transferred to the recording chamber and continuously perfused with recording ACSF with the following composition (in mM): 124 NaCl, 2.5 KCl, 1.2 NaH_2_PO_4_, 24 NaHCO_3_, 5 HEPES, 12.5 Glucose, 2 MgSO_4_ and 2 CaCl_2_. The flow rate of perfusion is about 2 ml/min. Only two brain slices per animal were used. Only one neuron was recorded for each slice. Each new experimental protocol used a fresh slice. Numbers in the manuscript represent the number of neurons tested for each parameter.

### Electrophysiology

The different nuclei of amygdala are distinguished under the microscope easily, as indicated by Fu *et al*.^13^. Neurons used for recording in BLA of left hemisphere were visualized using infrared differential interference contrast (IR-DIC) video microscopy with a 40x magnification water-immersion objective (BX51WI, Olympus). Patch electrodes (4–8 MΩ tip resistance) were made using a Flaming/Brown P-97 micropipette puller (Sutter Instruments Co), from borosilicate glass capillaries. The internal solution of the electrodes for recording excitatory post-synaptic currents (EPSCs) and action potentials (APs) contained (in mM): 133 K-gluconate, 8 NaCl, 0.6 EGTA, 10 HEPES, 2 Mg-ATP, and 0.3 Na-GTP, pH adjusted to 7.2–7.3 with KOH. After giga ohm (GΩ) seals (usually > 4 GΩ) were formed and the whole-cell configuration was obtained, neurons were tested if the resting membrane potential was more negative than −50 mV and direct depolarizing current injections evoked APs overshooting 0 mV when recording EPSC and APs. Since majority of neurons in BLA are excitatory neurons, we only included data of excitatory neurons according to the electrophysiological characteristics described by Washburn and Moises in response to intracellular injection of a depolarizing current (100~300 pA, step 50 pA, duration 1000 ms) in further analyses of sEPSC^56^,^57^. These neurons function as excitatory projection neurons in BLA^56^,^57^. The internal solution for recording sIPSCs contained (in mM): 140 CsCl, 10 HEPES, 1 EGTA, 2 MgCl_2_, 2Na-ATP, 0.3 Na-GTP, 5 QX314, pH was adjusted to 7.2 with CsOH. CNQX (10 μM) and D-AP5 (30 μM) were used in external solution to block excitatory synaptic transmission when recording sIPSCs. The holding potentials were −70 mV for recording EPSCs and IPSCs. Picrotoxin (100 μM) and CGP52432 (2 μM) were used in some experiment to block GABAergic inhibitory synaptic transmission. Both mEPSCs and mIPSCs were recorded in the presence of TTX (1 μM) in the extracellular solution. Capsaicin or CZP (both 5 μM) was applied to activate or to block TRPV1 receptors, respectively. All drugs were dissolved in ACSF on the day of experiment and added by perfusion.

Data were acquired using a Digidata 1440 A interface, MultiClamp 700B amplifier, and pClamp10 software. Data were sampled and filtered at 10 kHz with Bessel filter of amplifier. To ensure high-quality recordings, series resistance (<20 MΩ) was checked using membrane test function of pClamp10 software throughout the experiment. Data were stored on a computer and analyzed offline.

### Histology

At the end of behavioral experiment, rats where deeply anesthetized with chloral hydrate and perfused transcardially with 0.9% isotonic saline followed by 4% paraformaldehyde (PFA). Then the brains were post fixed overnight in PFA. After that, the brains were transferred to a 20% sucrose solution followed by a 30% sucrose solution. Brains were frozen and cut into 30 μm sections. Each section was mounted on slides and stained with cresyl violet staining solution (Beyotime Biotechnology, China) following Nissl staining method. A stereomicroscope (Olympus SZ61) combining with ISCapture software (Tucsen company, China) was used for placement verification of injection sites. The approximate site of injection was estimated for a given rat and mapped onto the corresponding drawing of rat brain atlas as filled black circles. If a site was not within the left BLA, data of the rat was not included in the analysis.

### Immunofluorescence study

The control or NMD rats were deeply anesthetized with chloral hydrate and perfused transcardially with 0.9% isotonic saline followed by 4% paraformaldehyde (PFA) in saline. Then the brains were post fixed overnight in PFA. After that, the brains were transferred to a 20% sucrose solution followed by a 30% sucrose solution. For labeling, 12 μm sections of BLA were simultaneously incubated with synaptophysin (1:100, ab8049, Abcam) and TRPV1R (1:200, ACC-030, Alomone labs) antibodies for overnight at 4 °C and then incubated with secondary antibody with Alexa Fluor 488 and 594 for 2 hours at room temperature. Negative controls were performed without the primary antibody.

### Data analyses

A fixed length of traces (3 min) was analyzed using MiniAnalysis program 6.0.3 (Synaptosoft) for frequency and amplitude distributions of miniature and spontaneous post-synaptic currents. Each detected event was visually checked to exclude the false data after the automatical detection for peaks. The detection threshold for an event in a set of data was set to three to four times the root mean square value of its background noise. Cumulative fractions were calculated before and after an addition of a drug. Statistical analysis for postsynaptic currents was performed using Kolmogorov-Smirnov test. All values were shown as mean ± SEM; error bars in the figures stand for SEM. Normality was checked for all data before comparisons using two-sample t-test, paired sample wilcoxon signed rank test, One-way ANOVA followed by Tukey post hoc test and Kruskal-Wallis ANOVA followed by Mann-Whitney test as post hoc analysis using Origin 8 (Origin Lab Inc., USA), as appropriate. Significance was set at *P* < 0.05.

## Additional Information

**How to cite this article**: Xiao, Y. *et al*. TRPV1-mediated presynaptic transmission in basolateral amygdala contributes to visceral hypersensitivity in adult rats with neonatal maternal deprivation. *Sci. Rep*. **6**, 29026; doi: 10.1038/srep29026 (2016).

## References

[b1] Greenwood-Van Meerveld B. . Stereotaxic delivery of corticosterone to the amygdala modulates colonic sensitivity in rats. Brain Res 893, 135–142 (2001). 11223001 10.1016/s0006-8993(00)03305-9

[b2] Drossman D. A., Camilleri M., Mayer E. A. & Whitehead W. E. AGA technical review on irritable bowel syndrome. Gastroenterology 123, 2108–2131 (2002). 12454866 10.1053/gast.2002.37095

[b3] Sandler R. S., Drossman D. A., Nathan H. P. & McKee D. C. Symptom complaints and health care seeking behavior in subjects with bowel dysfunction. Gastroenterology 87, 314–318 (1984). 6735075

[b4] Li L. . Upregulation of cystathionine beta-synthetase expression by nuclear factor-kappa B activation contributes to visceral hypersensitivity in adult rats with neonatal maternal deprivation. Mol Pain 8, 89 (2012). 23249427 10.1186/1744-8069-8-89 PMC3545973

[b5] Hu S. . Neonatal maternal deprivation sensitizes voltage-gated sodium channel currents in colon-specific dorsal root ganglion neurons in rats. Am J Physiol Gastrointest Liver Physiol 304, G311–321 (2013). 23139220 10.1152/ajpgi.00338.2012

[b6] Luo J. L. . Enhanced excitability and down-regulated voltage-gated potassium channels in colonic drg neurons from neonatal maternal separation rats. J Pain 12, 600–609 (2011). 21296029 10.1016/j.jpain.2010.11.005

[b7] Mulak A. & Bonaz B. Irritable bowel syndrome: a model of the brain-gut interactions. Med Sci Monit 10, RA55-62 (2004). 15260348

[b8] Zheng Z. H., Zeng Y. T., Yang W. & Wu J. Y. Irritable bowel syndrome may be induced by decreased neuroplasticity. Neuroendocrinol Lett 35, 655–665 (2014). 25702292

[b9] Neugebauer V., Li W., Bird G. C. & Han J. S. The amygdala and persistent pain. Neuroscientist 10, 221–234 (2004). 15155061 10.1177/1073858403261077

[b10] Marinelli S. . Presynaptic facilitation of glutamatergic synapses to dopaminergic neurons of the rat substantia nigra by endogenous stimulation of vanilloid receptors. J Neurosci 23, 3136–3144 (2003). 12716921 10.1523/JNEUROSCI.23-08-03136.2003 PMC6742307

[b11] Tanimoto S., Nakagawa T., Yamauchi Y., Minami M. & Satoh M. Differential contributions of the basolateral and central nuclei of the amygdala in the negative affective component of chemical somatic and visceral pains in rats. Eur J Neurosci 18, 2343–2350 (2003). 14622196 10.1046/j.1460-9568.2003.02952.x

[b12] Mena N. B., Mathur R. & Nayar U. Amygdalar involvement in pain. Indian J Physiol Pharmacol 39, 339–346 (1995). 8582745

[b13] Fu Y. & Neugebauer V. Differential mechanisms of CRF1 and CRF2 receptor functions in the amygdala in pain-related synaptic facilitation and behavior. J Neurosci 28, 3861–3876 (2008). 18400885 10.1523/JNEUROSCI.0227-08.2008 PMC2557030

[b14] Johnson A. C., Tran L. & Greenwood-Van Meerveld B. Knockdown of corticotropin-releasing factor in the central amygdala reverses persistent viscerosomatic hyperalgesia. Transl Psychiatry 5, e517 (2015). 25734510 10.1038/tp.2015.16 PMC4354346

[b15] Ji G., Li Z. & Neugebauer V. Reactive oxygen species mediate visceral pain-related amygdala plasticity and behaviors. Pain 156, 825–836 (2015). 25734993 10.1097/j.pain.0000000000000120 PMC4402250

[b16] Tache Y. Corticotrophin-releasing factor 1 activation in the central amygdale and visceral hyperalgesia. Neurogastroenterol Motil 27, 1–6 (2015). 25557223 10.1111/nmo.12495 PMC4389773

[b17] Myers B. & Greenwood-Van Meerveld B. Elevated corticosterone in the amygdala leads to persistent increases in anxiety-like behavior and pain sensitivity. Behav Brain Res 214, 465–469 (2010). 20573588 10.1016/j.bbr.2010.05.049

[b18] Davis M. Anatomic and physiologic substrates of emotion in an animal model. J Clin Neurophysiol 15, 378–387 (1998). 9821065 10.1097/00004691-199809000-00002

[b19] Phelps E. A. & LeDoux J. E. Contributions of the amygdala to emotion processing: from animal models to human behavior. Neuron 48, 175–187 (2005). 16242399 10.1016/j.neuron.2005.09.025

[b20] Walker D. L. & Davis M. Are fear memories made and maintained by the same NMDA receptor-dependent mechanisms? Neuron 41, 680–682 (2004). 15003167 10.1016/s0896-6273(04)00114-x

[b21] Hu S. . Sensitization of sodium channels by cystathionine beta-synthetase activation in colon sensory neurons in adult rats with neonatal maternal deprivation. Exp Neurol 248, 275–285 (2013). 23834820 10.1016/j.expneurol.2013.06.027

[b22] Diehl L. A. . Contextual Fear Conditioning in Maternal Separated Rats: The Amygdala as a Site for Alterations. Neurochem Res 39, 384–393 (2014). 24368626 10.1007/s11064-013-1230-x

[b23] Chung E. K., Bian Z. X., Xu H. X. & Sung J. J. Neonatal maternal separation increases brain-derived neurotrophic factor and tyrosine kinase receptor B expression in the descending pain modulatory system. Neurosignals 17, 213–221 (2009). 19546592 10.1159/000224631

[b24] Tominaga M. . The cloned capsaicin receptor integrates multiple pain-producing stimuli. Neuron 21, 531–543 (1998). 9768840 10.1016/s0896-6273(00)80564-4

[b25] Palazzo E., Rossi F. & Maione S. Role of TRPV1 receptors in descending modulation of pain. Mol Cell Endocrinol 286, S79–83 (2008). 18325659 10.1016/j.mce.2008.01.013

[b26] Chen K. . Blocking PAR2 attenuates oxaliplatin-induced neuropathic pain via TRPV1 and releases of substance P and CGRP in superficial dorsal horn of spinal cord. J Neurol Sci 352, 62–67 (2015). 25829079 10.1016/j.jns.2015.03.029

[b27] Giordano C. . TRPV1-dependent and -independent alterations in the limbic cortex of neuropathic mice: impact on glial caspases and pain perception. Cereb Cortex 22, 2495–2518 (2012). 22139792 10.1093/cercor/bhr328 PMC3464411

[b28] Fang D. . Interleukin-6-mediated functional upregulation of TRPV1 receptors in dorsal root ganglion neurons through the activation of JAK/PI3K signaling pathway: roles in the development of bone cancer pain in a rat model. Pain 156, 1124–1144 (2015). 25775359 10.1097/j.pain.0000000000000158

[b29] Zielinska M., Jarmuz A., Wasilewski A., Salaga M. & Fichna J. Role of transient receptor potential channels in intestinal inflammation and visceral pain: novel targets in inflammatory bowel diseases. Inflamm Bowel Dis 21, 419–427 (2015). 25437822 10.1097/MIB.0000000000000234

[b30] Zhu Y. . Nerve growth factor modulates TRPV1 expression and function and mediates pain in chronic pancreatitis. Gastroenterology 141, 370–377 (2011). 21473865 10.1053/j.gastro.2011.03.046 PMC7522725

[b31] Lapointe T. K. . TRPV1 sensitization mediates postinflammatory visceral pain following acute colitis. Am J Physiol Gastrointest Liver Physiol 309, G87–99 (2015). 26021808 10.1152/ajpgi.00421.2014

[b32] Zhou Q. . Decreased miR-199 augments visceral pain in patients with IBS through translational upregulation of TRPV1. Gut, gutjnl- 2013–306464 (2015). 10.1136/gutjnl-2013-306464 PMC4853572 25681400

[b33] Xu G. Y. . Transient receptor potential vanilloid 1 mediates hyperalgesia and is up-regulated in rats with chronic pancreatitis. Gastroenterology 133, 1282–1292 (2007). 17698068 10.1053/j.gastro.2007.06.015

[b34] Hong S. . Reciprocal changes in vanilloid (TRPV1) and endocannabinoid (CB1) receptors contribute to visceral hyperalgesia in the water avoidance stressed rat. Gut 58, 202–210 (2009). 18936104 10.1136/gut.2008.157594 PMC4236191

[b35] Qin H. Y. . Visceral hypersensitivity induced by activation of transient receptor potential vanilloid type 1 is mediated through the serotonin pathway in rat colon. Eur J Pharmacol 647, 75–83 (2010). 20826151 10.1016/j.ejphar.2010.08.019

[b36] Jurik A., Ressle A., Schmid R. M., Wotjak C. T. & Thoeringer C. K. Supraspinal TRPV1 modulates the emotional expression of abdominal pain. Pain 155, 2153–2160 (2014). 25139591 10.1016/j.pain.2014.08.012

[b37] Maione S. . Elevation of endocannabinoid levels in the ventrolateral periaqueductal grey through inhibition of fatty acid amide hydrolase affects descending nociceptive pathways via both cannabinoid receptor type 1 and transient receptor potential vanilloid type-1 receptors. J Pharmacol Exp Ther 316, 969–982 (2006). 16284279 10.1124/jpet.105.093286

[b38] de Novellis V. . The blockade of the transient receptor potential vanilloid type 1 and fatty acid amide hydrolase decreases symptoms and central sequelae in the medial prefrontal cortex of neuropathic rats. Mol Pain 7, 7 (2011). 21241462 10.1186/1744-8069-7-7 PMC3031241

[b39] Micale V. . Anxiolytic effects in mice of a dual blocker of fatty acid amide hydrolase and transient receptor potential vanilloid type-1 channels. Neuropsychopharmacology 34, 593–606 (2009). 18580871 10.1038/npp.2008.98

[b40] Zschenderlein C., Gebhardt C., von Bohlen Und Halbach O., Kulisch C. & Albrecht D. Capsaicin-induced changes in LTP in the lateral amygdala are mediated by TRPV1. PLoS One 6, e16116 (2011). 21249195 10.1371/journal.pone.0016116 PMC3020947

[b41] Wyllie D. J., Manabe T. & Nicoll R. A. A rise in postsynaptic Ca2+ potentiates miniature excitatory postsynaptic currents and AMPA responses in hippocampal neurons. Neuron 12, 127–138 (1994). 7507335 10.1016/0896-6273(94)90158-9

[b42] Okabe S., Miwa A. & Okado H. Spine formation and correlated assembly of presynaptic and postsynaptic molecules. J Neurosci 21, 6105–6114 (2001). 11487634 10.1523/JNEUROSCI.21-16-06105.2001 PMC6763142

[b43] Baas D., Aleman A. & Kahn R. S. Lateralization of amygdala activation: a systematic review of functional neuroimaging studies. Brain Res Brain Res Rev 45, 96–103 (2004). 15145620 10.1016/j.brainresrev.2004.02.004

[b44] Wager T. D., Phan K. L., Liberzon I. & Taylor S. F. Valence, gender, and lateralization of functional brain anatomy in emotion: a meta-analysis of findings from neuroimaging. Neuroimage 19, 513–531 (2003). 12880784 10.1016/s1053-8119(03)00078-8

[b45] Yang K., Kumamoto E., Furue H. & Yoshimura M. Capsaicin facilitates excitatory but not inhibitory synaptic transmission in substantia gelatinosa of the rat spinal cord. Neuroscience letters 255, 135–138 (1998). 9832191 10.1016/s0304-3940(98)00730-7

[b46] Nakatsuka T., Furue H., Yoshimura M. & Gu J. G. Activation of central terminal vanilloid receptor-1 receptors and αβ-methylene-ATP-sensitive P2X receptors reveals a converged synaptic activity onto the deep dorsal horn neurons of the spinal cord. The Journal of neuroscience 22, 1228–1237 (2002). 11850450 10.1523/JNEUROSCI.22-04-01228.2002 PMC6757570

[b47] Marinelli S., Vaughan C. W., Christie M. J. & Connor M. Capsaicin activation of glutamatergic synaptic transmission in the rat locus coeruleus *in vitro*. J Physiol-London 543, 531–540 (2002). 12205187 10.1113/jphysiol.2002.022863 PMC2290516

[b48] Tran L. & Keele N. B. CaMKIIalpha knockdown decreases anxiety in the open field and low serotonin-induced upregulation of GluA1 in the basolateral amygdala. Behav Brain Res 303, 152–159 (2016). 26821292 10.1016/j.bbr.2016.01.053 PMC4779371

[b49] Tran L., Chaloner A., Sawalha A. H. & Greenwood Van-Meerveld B. Importance of epigenetic mechanisms in visceral pain induced by chronic water avoidance stress. Psychoneuroendocrino 38, 898–906 (2013). 10.1016/j.psyneuen.2012.09.016 23084728

[b50] Banks S. J., Eddy K. T., Angstadt M., Nathan P. J. & Phan K. L. Amygdala–frontal connectivity during emotion regulation. Social cognitive and affective neuroscience 2, 303–312 (2007). 18985136 10.1093/scan/nsm029 PMC2566753

[b51] Huang Z. X. . Involvement of RVM-expressed P2X7 receptor in bone cancer pain: mechanism of descending facilitation. Pain 155, 783–791 (2014). 24447511 10.1016/j.pain.2014.01.011

[b52] Porreca F., Ossipov M. H. & Gebhart G. F. Chronic pain and medullary descending facilitation. Trends Neurosci 25, 319–325 (2002). 12086751 10.1016/s0166-2236(02)02157-4

[b53] Barreau F., Ferrier L., Fioramonti J. & Bueno L. Neonatal maternal deprivation triggers long term alterations in colonic epithelial barrier and mucosal immunity in rats. Gut 53, 501–506 (2004). 15016743 10.1136/gut.2003.024174 PMC1774003

[b54] Ren T. H. . Effects of neonatal maternal separation on neurochemical and sensory response to colonic distension in a rat model of irritable bowel syndrome. Am J Physiol Gastrointest Liver Physiol 292, G849–856 (2007). 17110521 10.1152/ajpgi.00400.2006

[b55] Ting J. T., Daigle T. L., Chen Q. & Feng G. In Patch-Clamp Methods and Protocols 221–242 (Springer, 2014). 10.1007/978-1-4939-1096-0_14 PMC4219416 25023312

[b56] Washburn M. S. & Moises H. C. Electrophysiological and morphological properties of rat basolateral amygdaloid neurons *in vitro*. J Neurosci 12, 4066–4079 (1992). 1403101 10.1523/JNEUROSCI.12-10-04066.1992 PMC6575963

[b57] Sah P., Faber E. S., Lopez De Armentia M. & Power J. The amygdaloid complex: anatomy and physiology. Physiol Rev 83, 803–834 (2003). 12843409 10.1152/physrev.00002.2003

